# Wharton's Jelly-Derived Mesenchymal Stem Cells with High Aurora Kinase A Expression Show Improved Proliferation, Migration, and Therapeutic Potential

**DOI:** 10.1155/2022/4711499

**Published:** 2022-04-11

**Authors:** Sun Jeong Kim, Sang Eon Park, Jang Bin Jeong, Shin Ji Oh, Alee Choi, Yun Hee Kim, Suk-joo Choi, Soo-young Oh, Gyu Ha Ryu, Hong Bae Jeon, Jong Wook Chang

**Affiliations:** ^1^Stem Cell Institute, ENCell Co. Ltd, Seoul 06072, Republic of Korea; ^2^Cell and Gene Therapy Institute, Samsung Medical Center, Seoul 06351, Republic of Korea; ^3^Department of Health Sciences and Technology, SAIHST, Sungkyunkwan University, Seoul 06351, Republic of Korea; ^4^Department of Obstetrics and Gynecology, Samsung Medical Center, Seoul 06351, Republic of Korea; ^5^Department of Medical Device Management and Research, SAIHST, Sungkyunkwan University School of Medicine, Seoul 06351, Republic of Korea; ^6^The Office of R&D Strategy & Planning, Samsung Medical Center, Seoul 06351, Republic of Korea

## Abstract

Mesenchymal stem cells (MSCs) are effective therapeutic agents that contribute to tissue repair and regeneration by secreting various factors. However, donor-dependent variations in MSC proliferation and therapeutic potentials result in variable production yields and clinical outcomes, thereby impeding MSC-based therapies. Hence, selection of MSCs with high proliferation and therapeutic potentials would be important for effective clinical application of MSCs. This study is aimed at identifying the upregulated genes in human Wharton's jelly-derived MSCs (WJ-MSCs) with high proliferation potential using mRNA sequencing. Aurora kinase A (AURKA) and dedicator of cytokinesis 2 (DOCK2) were selected as the upregulated genes, and their effects on proliferation, migration, and colony formation of the WJ-MSCs were verified using small interfering RNA (siRNA) techniques. mRNA expression levels of both the genes were positively correlated with the proliferation capacity of WJ-MSCs. Moreover, AURKA from human WJ-MSCs regulated the antiapoptotic effect of skeletal muscle cells by upregulating the chemokine (C motif) ligand (XCL1); this was further confirmed in the mdx mouse model. Taken together, the results indicated that AURKA and DOCK2 can be used as potential biomarkers for proliferation and migration of human WJ-MSCs. In particular, human WJ-MSCs with high expression of AURKA might have therapeutic efficacy against muscle diseases, such as Duchenne muscular dystrophy (DMD).

## 1. Introduction

Mesenchymal stem cells (MSCs) are multipotent stromal cells isolated from various tissues, including bone marrow, fat tissue, placenta, and Wharton's jelly (WJ). Since MSCs can maintain stemness, regulate self-renewal, differentiate into various cell types, and restore damaged tissues and cells, they have been used to treat various diseases [1–3].

Various proteins secreted from MSCs are known to have therapeutic potentials and play an important role in tissue repair. Proteins secreted by MSCs have shown therapeutic effects on diverse diseases, such as Alzheimer's disease and Duchenne muscular dystrophy (DMD) [4, 5]. Among the MSC-secreted proteins, the chemokine (C motif) ligand (XCL1) was reported to suppress apoptosis in skeletal muscle [6]. Based on such paracrine activities, WJ-MSCs have been suggested as effective therapeutic agents for various diseases, since they can control the effects of antiapoptosis, anti-inflammation, and immunomodulation via secretion of proteins [7, 8].

Although a sufficient number of MSCs would be required for their application in clinical trials, it is often difficult to achieve due to limitations, such as differences in proliferation capacity and production yield of MSCs across donors [9, 10]. MSCs with poor proliferation ability need prolonged culture to reach sufficient number; however, this inevitably induces cellular senescence, which potentially causes poor clinical outcomes [11]. Therefore, identification of MSC markers associated with high proliferative activity and therapeutic potential would be important for successful clinical application of MSCs. Early selection of WJ-MSCs with high proliferation ability might have positive clinical effects, since they can shorten the time to attain sufficient number of MSCs, avoiding long-term cultures that cause aging. Moreover, if the therapeutic effect can be predicted in advance, it can help clinical applications by reducing unnecessary effort or time. Therefore, early selection of cells with good treatment potential, using appropriate markers, can reduce the cost and shorten the time of attainment of MSCs, which would be beneficial for expanding MSCs under good manufacturing practice (GMP) conditions.

In this study, we identify proliferation-related factors in human WJ-MSCs; mRNA sequencing was performed to select the upregulated genes in WJ-MSCs with high proliferation potential, and Aurora kinase A (AURKA) and dedicator of cytokinesis 2 (DOCK2) were selected as the potential candidates.

## 2. Materials and Methods

### 2.1. Ethics Statement

This study was approved by the Institutional Animal Care and Use Committee of the Samsung Biomedical Research Institute (SBRI) at Samsung Medical Center. The SBRI complies with the Institute of Laboratory Animal Resources guidelines. The umbilical cords were collected from pregnant mothers with their prior consent, in accordance with the guidelines approved by the institutional review board of Samsung Medical Center (IRB#2016-07-102).

### 2.2. Cell Culture

WJ-MSCs were isolated and cultured according to the procedure described in a previous report [6]. For coculture experiments, mouse myoblast C2C12 cells (ATCC CRL-1772; American Type Culture Collection, Rockville, MD, USA) were seeded at 1 × 10^5^ cells/well in a 6-well plate, and apoptosis was induced by culturing them in serum-free medium for 24 h. WJ-MSCs were seeded at 1 × 10^5^ cells/insert in trans-well inserts (pore size 1 *μ*m, BD Biosciences, Franklin Lakes, NJ, USA) and cocultured with apoptosis-induced C2C12 cells. C2C12 cells were cultured with or without WJ-MSCs for 24 h under serum-starvation conditions.

### 2.3. mRNA Sequencing and Data Analysis

Total RNA was extracted using TRIzol reagent (Invitrogen, Waltham, MA, USA), and libraries were prepared therefrom using the SMARTer Stranded RNA-Seq Kit (Clontech Laboratories, Inc., CA, USA). High-throughput sequencing was performed as paired-end 100 sequencing using a HiSeq 2500 (Illumina, Inc., San Diego, CA, USA). Gene expression levels were evaluated according to Read Count (RC) using BEDTools [12], and normalization of the expression values was based on the quantile normalization method by edgeR within R [13]. Data mining and graphic visualization were conducted using the ExDEGA (E-biogen, Inc., Seoul, Korea) and MeV software. Distance was analyzed using the Euclidean distance metric, and linkage method was selected for average linkage clustering.

### 2.4. siRNA Transfection

All siRNAs were purchased from Bioneer Corporation (Daejeon, Korea); the sequences of AURKA-specific siRNA were (sense) 5′-GUGCAAUAACCUUCCUAGU-3′ and (antisense) 5′-ACUAGGAAGGUUAUUGCAC-3′ while those of DOCK2-specific siRNA were (sense) 5′-CUGAGAAUGACUUCCUAC-3′ and (antisense) 5′-UGUAGGAAGUCAUUCUCAG-3′. WJ-MSCs were grown in growth medium till 50% confluence and then transfected with siRNA using Lipofectamine RNAiMAX (Invitrogen) according to the manufacturer's protocol. All siRNAs were transfected at a final concentration of 25 nM in serum-free medium. The control used a nonsilencing scramble RNA (siNC) with at least four mismatches with any human, mouse, or rat gene.

### 2.5. Real-Time Quantitative Reverse Transcription Polymerase Chain Reaction (Real-Time qRT-PCR)

Total RNA was isolated from WJ-MSCs using an AccuPrep Universal RNA Extraction kit (Bioneer), according to the manufacturer's protocol. Real-time qRT-PCR was performed with a QuantStudio 6 Flex Real-Time PCR System (Thermo Fisher Scientific Inc., Waltham, MA, USA) and 2x Power SYBR Green Master Mix (Applied Biosystems, Waltham, MA, USA) using the following program: 95°C for 10 min, followed by 40 cycles of 95°C for 15 s, 56°C for 30 s, and 72°C for 30 s. Primers were purchased from Bioneer Corporation, and the primer sequences were as follows: AURKA, forward 5′-GGCCACTGAATAACACCCAAA-3′ and reverse 5′-AGAGGGCGACCAATTTCAAAG-3′; DOCK2, forward 5′-TTTCAACACCGTTCTGGAGG-3′ and reverse 5′-TCAGCGTTCTTAGGATTGGC-3′; and GAPDH, forward 5′-GAAGGTGAAGGTCGGAGT-3′ and reverse 5′-TGGCAACAATATCCACTTTACCA-3′. All PCR reactions were performed in triplicate, and comparative quantification of each target gene was normalized to GAPDH expression using the 2^-*ΔΔ*Ct^ method.

### 2.6. Western Blot Analysis

Cells were lysed with RIPA buffer (BIOSESANG, Sungnam, Korea) containing a protease inhibitor cocktail (AMRESCO, Solon, OH, USA) and incubated on ice for 20 min. Proteins were obtained via centrifugation at 15,000 × g for 30 min and separated using SDS-PAGE and transferred to membranes. The membranes were blocked with 5% skim milk at room temperature (RT) for 1 h and incubated overnight at 4°C with the following primary antibodies: anti-AURKA (Invitrogen), anti-p-AKT, anti-p-ERK, anti-XCL1 (R&D, Minneapolis, MN, USA), antiprotein kinase B (AKT), antiextracellular signal-regulated kinase (ERK), antipoly ADP ribose polymerase (PARP), anticleaved caspase-3, antifocal adhesion kinase (FAK), anti-p-FAK, anti-c-Jun N-terminal kinases (JNK), anti-p-JNK (Cell Signaling Technology, Danvers, MA, USA), antiannexin V, antifibronectin (Abcam, Cambridge, MA, USA), anti-DOCK2, and anti-*β*-actin (Santa Cruz Biotechnology, Dallas, TX, USA). After washing with TBST, the membranes were incubated with HRP-conjugated secondary antibodies at RT for 1 h. The membranes were then washed with TBST, and protein bands detected using a gel imaging system (Amersham Imager 600, GE Healthcare, Buckinghamshire, UK). Band intensities were measured using ImageJ (National Institutes of Health (NIH), Bethesda, MD, USA) and then normalized to that of *β*-actin.

### 2.7. Flow Cytometric Analysis

Harvested MSCs were washed in PBS and blocked with 2% FBS for 30 min at RT. WJ-MSCs were then incubated with the following antibodies for 20 min at RT in the dark: CD11b, CD14, CD19, CD34, CD44, CD45, CD73, CD90, CD105, CD166, and HLA-DR (BD Biosciences, Franklin Lakes, NJ, USA). At least 10,000 events were counted using the BD FACSVerse flow cytometer (BD Biosciences). Flow cytometry was performed for appropriate isotype controls as well.

### 2.8. Migration Assay

The migration of WJ-MSCs was estimated using a wound healing assay. WJ-MSCs were seeded at 1 × 10^5^ cells/well in 12-well plates and transfected with siRNA. They were incubated for 48 h and then treated with mitomycin C (10 *μ*g/ml, Sigma, St. Louis, MO, USA) in MEM-*α* without serum for 2 h. The center of cell monolayer was scratched using a 200 *μ*l pipette tip, and the wells were rinsed with the serum-free MEM-*α*; thereafter, cells were incubated with serum-free MEM-*α* for 24 h. Images were captured using a microscope (Olympus CKX41, Olympus, Tokyo, Japan). The area of wound was quantified by ImageJ. Migration capacity of WJ-MSCs was measured by calculating the percentage of wound closure [14]. Wound healing assays were analyzed using 15 fields for each group.

### 2.9. Clonogenic Assay

WJ-MSCs were seeded in 6-well plates at 1,000 cells/well. After incubation for 14 days, the WJ-MSCs were fixed with ice-cold 100% methanol and stained with 1% crystal violet solution (Sigma) for 30 min. Cells were then rinsed with distilled water, colonies with more than 50 cells were counted, and pictures of the plate were captured with a camera. Clonogenic assays were performed in triplicate, and each well had 1,000 cells to start with.

### 2.10. Cell Proliferation

Proliferation assays were performed at 0, 24, 72, and 144 h after seeding the transfected cells. After cells were seeded in 96-well plates at 2 × 10^3^ cells/well; cell proliferation was confirmed using the Cell Counting kit-8 (CCK-8, Dojindo, Tokyo, Japan), according to the manufacturer's instructions. OD values were measured at 450 nm using a microplate reader (xMark™ Microplate Absorbance Spectrophotometer, Bio-Rad Laboratories, Inc., Hercules, CA, USA). To determine the number of cells, cells were seeded in 12-well plates at 3 × 10^3^ cells/cm^2^ in triplicate. WJ-MSC proliferation was assessed by the doubling time and fold change.

### 2.11. Live/Dead Staining

Apoptosis was detected using the Apoptosis/Necrosis Detection Kit (Abcam) according to the manufacturer's instructions. Apoptotic cells were stained with Apopxin Green Indicator, and viable cells were stained with CytoCalcein Violet 450. Images were acquired using a microscope (Olympus IX51, Olympus) and analyzed using ImageJ.

### 2.12. Animals

Mdx (C57BL/10ScSn-Dmdmdx/J(mdx) (JAX#001801)) and normal control (C57BL/10ScSnJ (JAX#000476)) mice were purchased from Jackson Laboratory (Bar Harbor, ME, USA). Two- to five-month-old mdx mice were injected with 5 × 10^4^ WJ-MSCs in 100 *μ*l of PBS via the tail vein. Seven days after injection, the mice were sacrificed using isoprene.

### 2.13. Immunohistochemistry and Sirius Red Staining

The gastrocnemius muscles of mdx mice were harvested, fixed with 4% paraformaldehyde, and sectioned into paraffin blocks of 4 *μ*m thickness. IHC using annexin V was performed to confirm apoptosis. Tissue samples were incubated with annexin V antibody (Abcam), followed by incubation with goat anti-rabbit IgG secondary antibody (Alexa Fluor® 594 AffiniPure, Thermo Fisher Scientific) and counter-staining with Hoechst 33342 (Thermo Fisher Scientific). Fluorescence images were acquired using an LSM 700 confocal microscope (Carl Zeiss Meditec, Jena, Germany). Relative intensity of annexin V was calculated by dividing the intensity of annexin V fluorescence by the number of nuclei. Sirius Red staining was performed to observe muscle fibrosis, in accordance with standard procedures. Stained images were obtained under a ScanScope AT (Leica Microsystems, Buffalo Grove, IL, USA) and analyzed using ImageJ.

### 2.14. An Alu-Based Real-Time PCR

Genomic DNA was isolated from the gastrocnemius and thigh muscles of mdx mice using a Gentra Puregene Tissue kit (Qiagen Inc., Valencia, CA, USA), according to the manufacturer's protocol. Real-time PCR was performed under the following conditions: 95°C for 10 min, followed by 40 cycles of 95°C for 15 s and 68°C for 1 min. The forward and reverse primers for Alu were 5′-GTCAGGAGATCGAGACC ATCCC-3′ and 5′-TCCTGCCTCAGCCTCCCAAG-3′, respectively [15]. All experiments were conducted in triplicate using standards mixed with human DNA extracted from human MSCs and mouse DNA extracted from the liver of mdx mice.

### 2.15. Statistical Analysis

All data are shown as means ± standard error of the mean (SEM) of three or more replicates. Statistical comparisons between two groups were performed by two-tailed Student's *t*-test, and multiple comparisons across more than two groups were analyzed by one-way analysis of variance (ANOVA) with Tukey's or Dunnett's multiple-comparison post hoc test or Duncan's multiple range test. Differences were considered statistically significant at *p* < 0.05. Correlation coefficient (*r*) was measured using Pearson's correlation analysis. The SPSS Statistics 23 software (IBM Corp., Armonk, NY, USA) was used for all analyses.

## 3. Results

### 3.1. AURKA and DOCK2 Were Significantly Upregulated in WJ-MSCs with High Proliferation Capability

**Figure 1:**
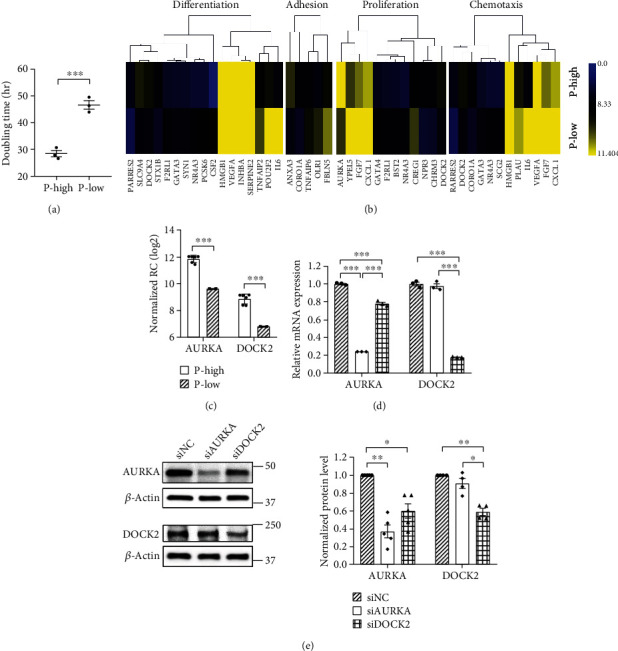
Comparative analysis of gene expression profiles of WJ-MSCs according to their proliferative capacity. (a) Doubling time of WJ-MSCs was measured, and the cells were classified into the proliferation-high (P-high) and proliferation-low (P-low) groups. (b) Heat map shows 33 differentially expressed genes related to cell differentiation, secretion, cell proliferation, cell adhesion, chemotaxis, and cell growth in both groups. (c) AURKA and DOCK2 genes expressed more significantly in the P-high group than in the P-low group. DEG selection criteria: significant fold changes (*p* < 0.05), fold change > 3, normalized read count (RC) > 6. (d) mRNA expression levels of AURKA and DOCK2 decreased at 48 h after siRNA transfection. (e) Suppression of AURKA and DOCK2 expression was examined by western blotting at 72 h after siRNA transfection. Normalized protein level indicates the value converted based on the siNC group. Data are shown as means ± SEM. Two-tailed Student's *t*-test (a, c). One-way ANOVA with Tukey's multiple comparisons test (d, e). ^∗∗∗^*p* < 0.001, ^∗∗^*p* < 0.01, and ^∗^*p* < 0.05.

To divide WJ-MSCs into two groups according to their proliferative capacity, we estimated the doubling time of WJ-MSCs and classified them into a proliferation-high (P-high) group and a proliferation-low (P-low) group [16]. The doubling time was significantly shorter in the P-high group (28.4 ± 1.2 h) than in the P-low group (46.5 ± 1.6 h) (Figure 1(a)).

To confirm the difference in mRNA levels between the groups, mRNA sequencing was performed using the RNA isolated from each group. Heat map indicated the mean of 33 differentially expressed genes related to cell differentiation, cell proliferation, cell growth, cell adhesion, and chemotaxis in the P-high group compared to that in the P-low group (Figure 1(b)). Upregulated genes in the P-high group (compared to those in the P-low group) included coronin-1A (CORO1A), syntaxin-1B (STX1B), high mobility group box 1 (HMGB1), DOCK2, AURKA, solute carrier family 9, subfamily A (SLC9A4), cholinergic/acetylcholine receptor M3 (CHRM3), natriuretic peptide receptor C/guanylate cyclase C (NPR3), retinoic acid receptor responder protein 2 (RARRES2), and annexin A3 (ANXA3). AURKA and DOCK2 were selected for further experiments, since their mRNA levels were significantly higher in the P-high group than in the P-low group (*p* < 0.05) (Figure 1(c)).

To confirm the role of AURKA and DOCK2 in WJ-MSCs, we suppressed the expression of these genes via transfection with siAURKA and siDOCK2. Real-time qRT-PCR and western blotting were performed thereafter to confirm whether the expression of AURKA and DOCK2 had indeed decreased (Figures 1(d) and 1(e)). Results indicated that siRNA transfection effectively knocked down AURKA and DOCK2 in WJ-MSCs. Flow cytometric analysis was performed to determine whether siRNA transfection affected the stemness of WJ-MSCs. As shown in Supplementary Figure 1, knockdown of AURKA and DOCK2 had no effect on the stemness of WJ-MSCs.

### 3.2. Knockdown of AURKA and DOCK2 Inhibited Proliferation and Migration of WJ-MSCs

**Figure 2:**
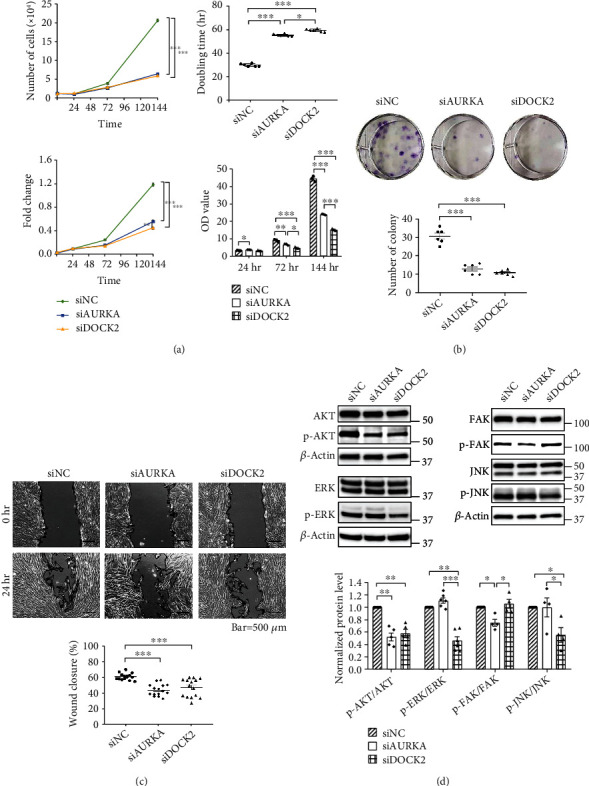
Knockdown of AURKA and DOCK2 inhibited cell proliferation, colony formation, and migration of WJ-MSCs. (a) Proliferation was evaluated by cell counting and CCK-8 assay at 0, 24, 72, and 144 h after seeding the siRNA-transfected cells. (b) Colony formation assays were performed in triplicate, with 1,000 cells per well to start with. Colonies were stained and counted two weeks after plating. (c) Migration was examined by wound healing assay. Knockdown of AURKA and DOCK2 inhibited the migration of WJ-MSCs. (d) Downregulation of AURKA inhibited phosphorylation of AKT and FAK while that of DOCK2 inhibited phosphorylation of AKT, ERK, and JNK. Normalized protein level indicates the value converted based on the siNC group. Data are shown as means ± SEM. One-way ANOVA with Dunnett's multiple comparison test (b–d), and Tukey's multiple comparisons test (a). ^∗∗∗^*p* < 0.001, ^∗∗^*p* < 0.01, and ^∗^*p* < 0.05.

Since AURKA and DOCK2 are known to regulate cell proliferation and migration [17–19], we confirmed their functions through gene suppression using siRNA transfection. To confirm whether knockdown of AURKA and DOCK2 decreased the proliferation of WJ-MSCs, cell counting and CCK-8 assays were performed at 0, 24, 72, and 144 h after seeding the transfected cells. Doubling time was 31.1 ± 0.4 h in the siNC-transfected group (control), 55.3 ± 0.8 h in the siAURKA-transfected group, and 59.2 ± 0.8 h in the siDOCK2-transfected group, hence suggesting that suppression of AURKA and DOCK2 increased the doubling time of WJ-MSCs. Fold changes were calculated by dividing each OD value by that at 0 h. Compared to the siNC-transfected group, the siAURKA- and siDOCK2-transfected groups showed significantly decreased cell proliferation over time, the effect being notable in the siDOCK2-transfected group (Figure 2(a)). Clonogenic assays were performed for two weeks to confirm whether AURKA and DOCK2 affected cell survival over a prolonged period of time. The plating efficiency of WJ-MSCs used in this experiment was 3.9%, and clonogenicity of the siAURKA- and siDOCK2-transfected groups was significantly reduced compared to that in the siNC-transfected group (*p* < 0.001) (Figure 2(b)). Degree of wound closure was significantly decreased in the siAURKA-transfected group and siDOCK2-transfected group compared to that in the siNC-transfected group (*p* < 0.001). Downregulation of AURKA and DOCK2 inhibited the migration of WJ-MSCs (Figure 2(c)). We performed western blotting to identify the effect of AURKA and DOCK2 on the phosphorylation of AKT, ERK, FAK, and JNK, which are involved in migration and cell proliferation [20–24]. AURKA knockdown decreased the phosphorylation of AKT and FAK while DOCK2 knockdown decreased the phosphorylation of AKT, ERK, and JNK (*p* < 0.05) (Figure 2(d)). Collectively, the results demonstrated that AURKA affects colony formation, migration, and cell proliferation through the AURKA/AKT/FAK signaling pathway while DOCK2 affects the same through AKT, ERK, and JNK.

### 3.3. mRNA Expression Levels of AURKA and DOCK2 Were Significantly Negatively Correlated with Doubling Time of WJ-MSCs

**Figure 3:**
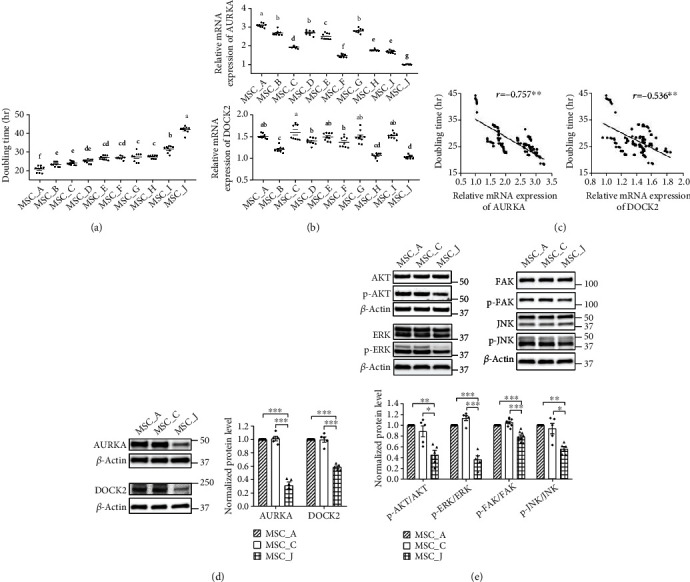
Correlation between doubling time and mRNA expression of AURKA and DOCK2 in WJ-MSCs. (a) The graph shows doubling time in ten lots of WJ-MSCs. (b) Real-time qRT-PCR analysis showed mRNA expression levels of AURKA and DOCK2 in WJ-MSCs. Relative mRNA expression indicates the value converted based on that of MSC_J. Different letters above bars mean significant difference from others (one-way ANOVA followed by Tukey's multiple range test, *p* < 0.05). (c) Pearson's correlation analysis confirmed a strong negative relationship between mRNA expression levels of AURKA and doubling time across the lots of WJ-MSCs. mRNA expression levels of DOCK2 and doubling time of WJ-MSCs showed a moderate negative relationship. (d) Western blotting of the protein levels of AURKA and DOCK2 in three WJ-MSCs. (e) Western blotting of the phosphorylation level of AKT, ERK, FAK, and JNK in three WJ-MSCs. Normalized protein level indicates the value converted based on that of MSC_A. Data are shown as means ± SEM. One-way ANOVA with Tukey's multiple comparisons test (a, b) and Dunnett's multiple comparisons test (d, e). ^∗∗∗^*p* < 0.001, ^∗∗^*p* < 0.01, and ^∗^*p* < 0.05.

We checked the doubling time (Figure 3(a)) and mRNA expression levels of AURKA and DOCK2 genes in WJ-MSCs isolated from ten donors (Figure 3(b)) and verified the correlation between mRNA expression levels of these genes and doubling time through Pearson's correlation analysis (Figure 3(c)). The doubling time showed a strong negative correlation with mRNA expression levels of AURKA (*r* = −0.757, *p* < 0.01) and a moderate negative correlation with those of DOCK2 (*r* = −0.536, *p* < 0.01) [25] (Figure 3(c)). Together, the results suggest that AURKA and DOCK2 are associated with proliferation of WJ-MSCs.

Based on the above results, we selected three WJ-MSCs to confirm the protein levels of AURKA and DOCK2 and phosphorylation of AKT, ERK, FAK, and JNK; MSC_A had the highest expression of AURKA, MSC_C had the highest expression of DOCK2, and MSC_J had the lowest expression of both the genes. MSC_A and MSC_C showed no difference in the protein expression of AURKA and DOCK2. On the other hand, the protein expression of AURKA and DOCK2 decreased prominently in MSC_J compared to that in MSC_A and MSC_C (*p* < 0.001) (Figure 3(d)). Similarly, although the phosphorylation of AKT, ERK, FAK, and JNK did not differ between MSC_A and MSC_C, it decreased significantly in MSC_J than in both (*p* < 0.05) (Figure 3(e)). The results suggested that AURKA and DOCK2 affect the phosphorylation of kinases associated with proliferation and migration of WJ-MSCs.

### 3.4. WJ-MSCs with High Expression of AURKA and DOCK2 Were More Effective in Inhibiting Apoptosis of C2C12 Cells than Those with Low Expression of Both

**Figure 4:**
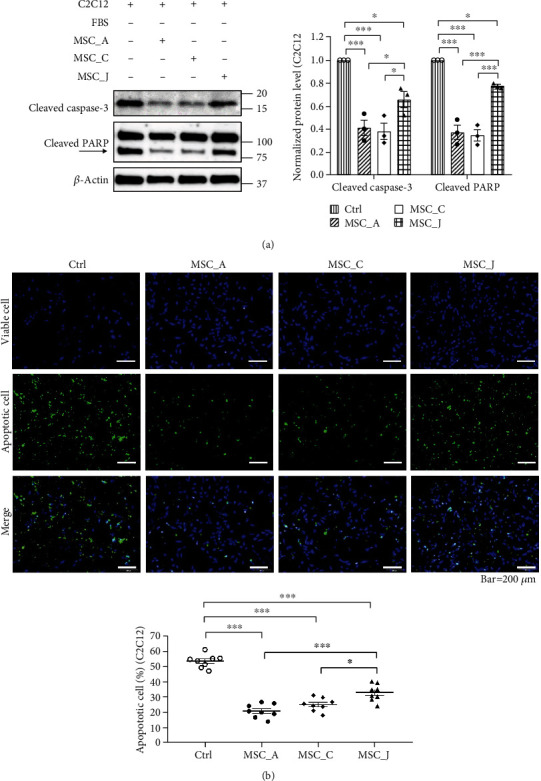
WJ-MSCs with high expression of AURKA and DOCK2 were more effective in reducing apoptosis of mouse skeletal myoblast cells (C2C12). (a) Inhibition of apoptosis was evaluated by protein expression levels of cleaved caspase-3 and cleaved PARP in apoptosis-induced C2C12 cells after coculture with WJ-MSCs. Normalized protein level indicates the value converted based on that of the control group. (b) Representative fluorescence images showed antiapoptotic effect of WJ-MSCs in apoptosis-induced C2C12 (green, apoptotic cells; blue, viable cells). The chart quantified the apoptotic cell rate of each group. Data are shown as means ± SEM. One-way ANOVA with Tukey's multiple comparisons test. ^∗∗∗^*p* < 0.001, ^∗∗^*p* < 0.01, and ^∗^*p* < 0.05.

An apoptosis induction model was established in vitro to verify whether AURKA and DOCK2 are involved in therapeutic effects. Degree of apoptosis was determined by the expression of apoptosis marker proteins and fluorescence staining of apoptotic cells; apoptosis was induced in C2C12 cells cultured in serum-starved medium compared to that in serum-containing medium (Supplementary Figure 2). Next, the apoptosis-induced C2C12 cells and WJ-MSCs were cocultured to determine whether the antiapoptotic effect varied depending on the mRNA levels of AURKA and DOCK2. Antiapoptotic effects were compared using MSC_A with the highest expression of AURKA, MSC_C with the highest expression of DOCK2, and MSC_J with the lowest expression of both the genes. In apoptosis-induced C2C12 cells cocultured with MSC_A or MSC_C, the expression of cleaved caspase-3 and cleaved PARP decreased remarkably (*p* < 0.001) compared to that in apoptosis-induced C2C12 cells (control). However, in apoptosis-induced C2C12 cells cocultured with MSC_J, expression of cleaved caspase-3 and cleaved PARP decreased gently compared to that in the control group (*p* < 0.05) and increased significantly compared to that in the MSC_A and MSC_C groups (cleaved caspase-3, *p* < 0.05; cleaved PARP, *p* < 0.001) (Figure 4(a)). Apoptosis kits were used to identify the apoptotic rate among the total cells, where viable cells were stained blue and apoptotic cells were stained green. Fluorescence images were representative of the apoptosis-induced C2C12 cells 24 h after coculture with WJ-MSCs. Apoptotic rate in the control group was 53.42 ± 1.52% while that significantly decreased in all groups of C2C12 cocultured with MSCs (*p* < 0.001). However, among MSCs, apoptotic rate in the MSC_J groups increased significantly compared to that in the MSC_A and MSC_C groups (*p* < 0.05) (Figure 4(b)). These results showed MSC_J to be less effective in suppressing apoptosis of C2C12 cells compared to MSCs _A and MSC_C.

### 3.5. Knockdown of AURKA Suppressed Antiapoptotic Effect of WJ-MSCs through Reduction of XCL1 Protein Expression

**Figure 5:**
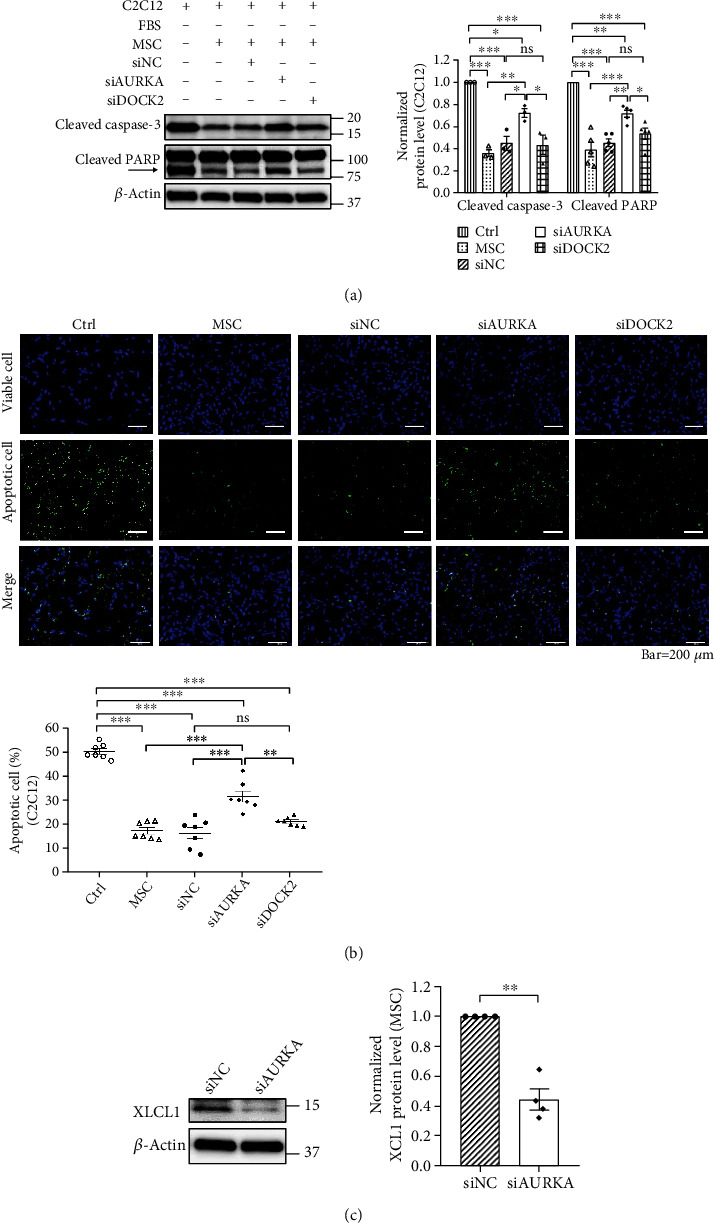
Knockdown of AURKA inhibited antiapoptotic effect of WJ-MSCs and was accompanied by the reduction of XCL1 protein. (a) Western blotting of the protein expression levels of cleaved caspase-3 and cleaved PARP in apoptosis-induced C2C12 cells. Normalized protein level indicates the value converted based on that of the control group. (b) Representative fluorescence images showed whether the suppression of AURKA and DOCK2 inhibited antiapoptotic effects of WJ-MSC in C2C12 (green, apoptotic cells; blue, viable cells). (c) Western blotting of the expression level of XCL1 protein in siAURKA-transfected WJ-MSCs. Normalized protein level indicates the value converted based on that of the siNC group. Data are shown as means ± SEM. One-way ANOVA with Tukey's multiple comparisons test (a, b). Two-tailed Student's *t*-test (c). ^∗∗∗^*p* < 0.001, ^∗∗^*p* < 0.01, and ^∗^*p* < 0.05.

To directly determine whether the antiapoptotic effect of WJ-MSCs on skeletal muscle cells could be related to the expression of AURKA and DOCK2, we suppressed AURKA and DOCK2 through siRNA transfection. After apoptosis-induced C2C12 cells (control) were cultured with WJ-MSCs, siNC-transfected WJ-MSCs, siAURKA-transfected WJ-MSCs, and siDOCK2-transfected WJ-MSCs, western blotting was performed to confirm the protein expression of cleaved caspase-3 and cleaved PARP. The apoptosis-induced C2C12 cells cocultured with siDOCK2-transfected WJ-MSCs showed no significant difference in the expression of apoptotic markers compared to the siNC-co-cultured group. Meanwhile, the expression of cleaved caspase-3 and cleaved PARP significantly increased in the apoptosis-induced C2C12 groups cocultured with siAURKA-transfected WJ-MSCs compared to that in both the siNC-co-cultured group and the siDOCK2-co-cultured group (*p* < 0.05) (Figure 5(a)). Fluorescence images were representative of the apoptosis-induced C2C12 cells after coculture with WJ-MSCs. Similar to western blotting results, apoptotic rate in apoptosis-induced C2C12 cocultured with WJ-MSCs decreased compared to that in the control group (*p* < 0.001). However, the apoptotic rate increased significantly in the siAURKA group compared to the siNC group and siDOCK2 group (*p* < 0.01) (Figure 5(b)). When AURKA was knocked down in WJ-MSCs, both protein expression of apoptosis markers and apoptotic rate were significantly increased in apoptosis-induced C2C12 cells than in both the siNC group and siDOCK2 group. Results indicated that the antiapoptotic effect of WJ-MSCs on skeletal muscle cells is affected by AURKA.

Our previous study had shown that XCL1 secreted by WJ-MSCs prevented the apoptosis of C2C12 [6]. Therefore, we investigated whether AURKA in WJ-MSCs could be related to an antiapoptotic effect, when cocultured with C2C12 cells, via XCL1. Expression of XCL1 protein in the siAURKA-transfected group was reduced (*p* < 0.01) compared to that in the siNC-transfected group (Figure 5(c)), indicating that AURKA inhibits apoptosis by upregulating XCL1 protein in skeletal muscle cells.

### 3.6. Expression Levels of AURKA Were Related to Antiapoptotic Effect of WJ-MSCs on Skeletal Muscle of the mdx Mouse

**Figure 6:**
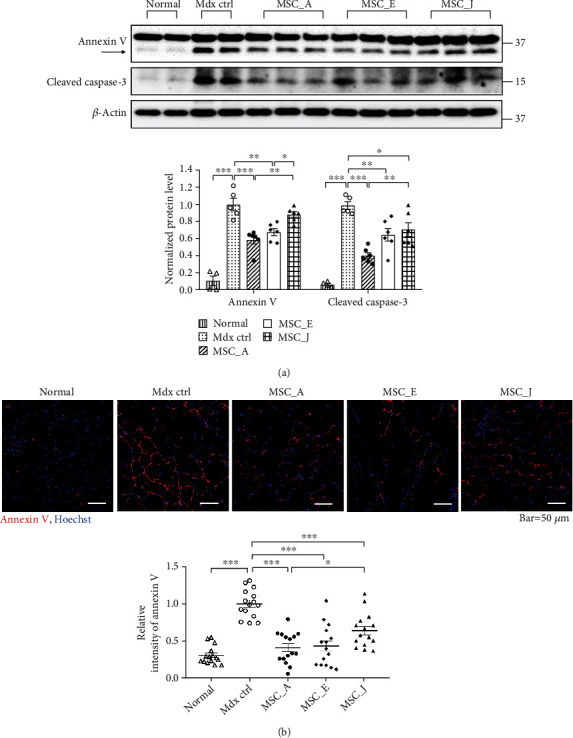
Antiapoptotic effect based on the differences in AURKA mRNA expression of WJ-MSCs in the skeletal muscle of mdx mice. (a) Protein expression levels of annexin V and cleaved caspase-3 were confirmed using western blotting. (b) Immunohistochemistry of annexin V was conducted in the gastrocnemius muscles of mdx mice (red, annexin V; blue, Hoechst). Relative intensity of annexin V indicates the value converted based on that of the mdx control group. Data are shown as means ± SEM. One-way ANOVA with Tukey's multiple comparison test (a) and Dunnett's multiple comparison test (b). ^∗∗∗^*p* < 0.001, ^∗∗^*p* < 0.01, and ^∗^*p* < 0.05.

Since the antiapoptotic effect of WJ-MSCs is influenced by AURKA and the difference in mRNA levels of DOCK2 between donors is only slight, we selected WJ-MSCs based on the mRNA level of AURKA and conducted subsequent animal experiments. To evaluate whether AURKA expression and proliferative capacity of WJ-MSCs were effective in suppressing apoptosis in DMD pathology, we confirmed the protein expression of annexin V using western blotting and immunohistochemistry in the gastrocnemius muscles of mdx mouse (a DMD model). Experiments were performed 7 days after intravenous (IV) injection of WJ-MSCs with different AURKA mRNA levels and proliferative capacity into the mice. MSC_A had the highest expression of AURKA and the shortest doubling time, whereas MSC_E had the median value of both AURKA expression and doubling time, and MSC_J had the lowest expression of AURKA and the longest doubling time. Compared to that in the mdx control mice, protein expression of annexin V was significantly decreased in MCS_ A- and MCS_E-injected mdx mice; however, there was no difference in MSC_J-injected mdx mice. Annexin V levels in MSC_J-injected mdx mice were higher than in MSC_A-injected mdx mice and MSC_E-injected mdx mice (*p* < 0.05). In MCS_ A-, MCS_E-, and MSC_J-injected mdx mice, the cleaved caspase-3 protein levels were significantly reduced compared to that in the mdx control mice. The level of cleaved caspase-3 in MSC_J-injected mdx mice was significantly higher than that in MSC_A-injected mdx mice (*p* < 0.01) (Figure 6(a)). IHC results were similar as above. In MSC-injected mdx mice, the relative intensity of annexin V fluorescence significantly decreased compared to that in mdx control mice (*p* < 0.001). The MSC_J-injected mdx mice showed a relatively stronger intensity of annexin V fluorescence than the MSC_A-injected mdx mice (*p* < 0.05) and MSC_E-injected mdx mice (*p* = 0.07) (Figure 6(b)), thereby indicating that WJ-MSCs, with low levels of AURKA expression, are ineffective in suppressing muscle cell death.

## 4. Discussion

The deviation among MSCs in terms of cell growth and yield, depending on donors, has been one of the major limitations in their application to cell therapy [9, 10]. Selection of MSCs with high proliferation capacity and therapeutic potency would be crucial in the initial stage of manufacturing MSCs as a medicinal product to resolve the donor variation. In this study, we classified WJ-MSCs isolated from various donors into two groups, namely, proliferation-high (P-high) and proliferation-low (P-low), based on their proliferation capacity, and performed gene expression profiling to examine the difference in gene expression between the two groups. We identified AURKA and DOCK2 as cell proliferation markers among the upregulated genes in the P-high group compared to that in the P-low group (Figure 1). A previous study comparing WJ-MSCs and BM-MSCs has reported that cell cycle-related genes, such as Aurora kinase B (AURKB), cyclin D2 (CCND2), and cell division cycle 25C (CDC25C), are involved in the proliferation capacity of WJ-MSCs [26]. Another study comparing the proliferation capacity of WJ-MSCs in hypoxia and normoxia showed growth factors, such as fibroblast growth factor-17 (FGF-17), vascular endothelial growth factor A (VEGFA), and insulin-like growth factor binding protein 3 (IGFBP3), to be involved in the proliferation of WJ-MSCs [27]. We identified novel factors related to the donor-dependent variation in proliferation capacity of WJ-MSCs by examining differential gene expression under the same conditions in WJ-MSCs originating from different donors.

**Figure 7:**
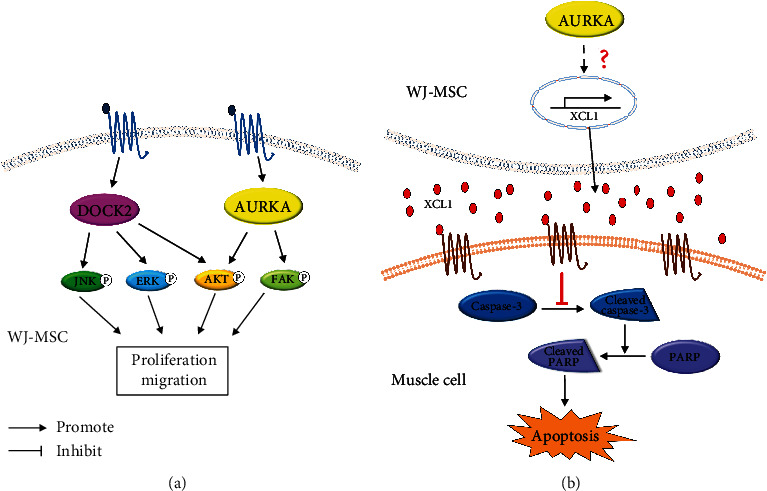
Both AURKA and DOCK2 contributed to cell proliferation and migration of WJ-MSCs, and AURKA of WJ-MSCs inhibited apoptosis in mdx mouse through XCL1. (a) In WJ-MSCS, both AURKA and DOCK2 promoted the proliferation and migration of WJ-MSCs through phosphorylation of kinases. (b) In WJ-MSCs, AURKA stimulated XCL1 transcription, causing XCL1 secretion from WJ-MSCs. The secreted XCL1 bound to the chemokine receptors of muscle cells, thereby inhibiting the cleavage of caspase-3 and PARP and suppressing the apoptosis of muscle cells.

Results demonstrated that the knockdown of AURKA and DOCK2 diminished both cell proliferation and migration rate; knockdown of AURKA reduced phosphorylation of AKT and FAK while that of DOCK2 reduced the phosphorylation of AKT, ERK, and JNK (Figure 2). These results indicated the involvement of AURKA and DOCK2 in cell proliferation and migration by activating AKT and FAK via AURKA and by activating AKT, ERK, and JNK via DOCK2 (Figure 7(a)). AURKA, a centrosome-associated serine/threonine kinase, has been shown to modulate essential mitotic events, including centrosome maturation, bipolar spindle assembly, and G2-M transition [28–30] and promote cell survival, migration, invasion, and proliferation in various cancer cells by activating AKT and FAK [17, 31, 32]. DOCK2, an atypical guanine nucleotide exchange factor (GEF), is involved in lymphocyte migration by regulating the actin cytoskeleton [19], as well as the motility and polarity during neutrophil chemotaxis [33] in immune cells. It also promotes cell proliferation, invasion, and migration by phosphorylation of AKT, ERK, and JNK in cancer cells [18, 34, 35]. Although AURKA and DOCK2 have been extensively reported to be closely related to cell proliferation and migration of cancer cells, the function of these two molecules in WJ-MSCs has not yet been studied extensively. In our current study, we demonstrated, to the best of our knowledge, for the first time that AURKA and DOCK2 regulate the proliferation and migration of human WJ-MSCs. In WJ-MSCs derived from ten different donors, the mRNA expression levels of AURKA and DOCK2 were significantly negatively correlated with doubling time (Figure 3(c)), which implied that AURKA and DOCK2 are potential biomarkers to identify and select the cells with high proliferation capacity in the initial stage of development of cell therapy to control the donor-dependent variation in medicinal products.

Among the factors determining the therapeutic potential of WJ-MSCs, enhanced migration and proliferation capacities definitely contribute more to the therapeutic effect of MSCs in various diseases [36–39]. Since AURKA and DOCK2 are involved in cell proliferation and migration, we applied in vitro apoptosis models to determine whether WJ-MSCs with high expression of both genes could be more effective in treating muscle disease. As expected, WJ-MSCs with high expression of AURKA and DOCK2 were more effective in suppressing apoptosis (Figure 4). However, knockdown results of both genes showed that the factor inhibiting apoptosis was AUKRA, not DOCK2, and AURKA regulated protein expression of XCL1, known to inhibit apoptosis [6] (Figure 5). We also demonstrated the anti-poptotic effect of AURKA in in vivo model. An enhanced antiapoptotic effect was observed in the limbs of mdx mice, a DMD mouse model, injected with WJ-MSCs highly expressing AURKA compared to that in mice injected with WJ-MSCs expressing AURKA at a low level (Figure 6). These findings indicated that AURKA in WJ-MSCs is not only involved in proliferation and migration but also in suppressing the apoptosis of skeletal muscle cells by regulating XCL1 expression. AKT and FAK have been reported to be involved in the transcription of chemokine genes [40, 41]; chemokines promote myoblast proliferation, which affects muscle repair [42, 43]. Therefore, we hypothesized that in WJ-MSCs, some kinases activated by AURKA might induce the transcription of XCL1, a family of chemokines; XCL1 protein is, hence, increased in cells, eventually promoting the secretion of XCL1 protein. It may then reach and bind to the chemokine receptors of muscle cells, thereby causing inhibition of apoptosis in the muscle tissue by reducing the cleavage of caspase-3 and PARP (Figure 7(b)).

Lee et al. [44] have demonstrated that MSCs with enhanced proliferation and migration due to ethionamide were retained longer when injected into mouse brain and were expected to show better therapeutic effect than naïve MSCs in the treatment of brain diseases. Therefore, WJ-MSCs with enhanced proliferation and migration, due to high expression of AURKA, probably showed improved apoptotic effect in the mdx mouse due to the higher number of WJ-MSCs retained in the muscle tissue than the WJ-MSCs with low expression of AURKA. In fact, the number of WJ-MSCs with low expression of AURKA, which remained in the limb muscle tissue of mdx mouse, was found to be less than that of WJ-MSCs highly expressing AURKA (Supplementary Figure 3(a)). In addition, the WJ-MSCs with low-level expression of AURKA showed less therapeutic effect on muscle fibrosis, a pathological hallmark of DMD [45], than those with high-level expression of AURKA. Not only expression of fibronectin, a fibrosis marker, but also accumulation of collagen was significantly reduced in the muscle tissue of mdx mice injected with MSCs with high-level expression of AURKA compared to that in mice injected with MSCs expressing low-level AURKA (Supplementary Figure 3(b), (c)). The results collectively suggested that MSCs highly expressing AURKA are more effective in ameliorating muscle fibrosis since the cells could migrate to limb muscle faster and stay in the muscle tissue longer.

In this study, expression of AURKA was found to regulate the capacity of cell proliferation and migration, demonstrating a treatment effect in DMD. Therefore, we proposed the mRNA expression level of AURKA to possibly be a useful standard for identifying and selecting WJ-MSCs for the development and manufacture of cell therapy products for DMD treatment. However, the downstream signaling pathways of AURKA, which are involved in the transcription of XCL1 in WJ-MSCs, are yet to be identified. Further studies on these would be required, and the factors regulating fibrosis in skeletal muscle would need to be identified.

## 5. Conclusions

This study was the first to confirm that AURKA and DOCK2 contribute to the proliferation and migration of WJ-MSCs. Especially, AURKA can be utilized as a strong potential selection marker for the proliferation of WJ-MSCs, and early selection of WJ-MSCs with it could not only reduce production costs but also enable large-scale production of WJ-MSCs for clinical applications. In addition, since AURKA mediated antiapoptosis processes of skeletal muscle cell by regulating XCL1, the selection of WJ-MSCs based on the level of AURKA could be an alternative option to improve their therapeutic efficacy in treating muscle diseases, such as DMD.

## Data Availability

The data used to support the findings of this study are available from the corresponding authors upon request.

## Supplementary Materials

Supplementary Materials Supplementary Figure 1: siRNA transfection of AURKA and DOCK2 in WJ-MSCs did not affect cell stemness. Stemness was evaluated using flow cytometry, by identifying the expression of MSC-positive markers (CD44, CD73, CD90, CD105, and CD166). Expression of MSC-negative markers was confirmed using the hematopoietic markers (CD11b, CD14, CD19, CD34, CD45, and HLA-DR). Supplementary Figure 2: C2C12 cells were incubated in serum-free medium for 24 h to cause apoptosis. (a) The C2C12 cells were cultured with or without FBS for 24 h. (b) The apoptosis markers were upregulated in C2C12 cultured without FBS. Normalized protein levels indicate the value converted based on the FBS (-) group. (c) Representative fluorescence images showed apoptosis induction due to serum deficiency in C2C12 (green, apoptotic cells; blue, viable cells). The chart quantified the apoptotic rate of each group. Data are shown as means ± SEM. Two-tailed Student's *t*-test. ^∗∗∗^*p* < 0.001 and ^∗∗^*p* < 0.01. Supplementary Figure 3: residual cell number and antifibrotic effect according to the differences in AURKA mRNA expression of WJ-MSCs in the skeletal muscle of mdx mice. (a) The number of residual WJ-MSCs in mouse leg muscle (gastrocnemius muscle and thigh muscle) was identified using human Alu using real-time PCR. (b) Protein expression levels of fibronectin were confirmed using western blot. Normalized protein level indicates the value converted based on the mdx control group. One-way ANOVA with Tukey's multiple comparison test. (c) Fibrotic area was confirmed based on collagen deposition through Sirius Red staining of the gastrocnemius muscles of mdx mice. Data are shown as means ± SEM. One-way ANOVA with Duncan's multiple range test (a) and Tukey's multiple comparisons test (b, c). ^∗∗∗^*p* < 0.001, ^∗∗^*p* < 0.01, and ^∗^*p* < 0.05.
