# Supplementary material for: Wharton's Jelly-Derived Mesenchymal Stem Cells with High Aurora Kinase A Expression Show Improved Proliferation, Migration, and Therapeutic Potential
Source: Stem Cells Int. 2022 Apr 11;2022:4711499. doi: 10.1155/2022/4711499 (PMC9017458; doi:10.1155/2022/4711499)
Supplement: Supplementary Materials — Supplementary Figure 1: siRNA transfection of AURKA and DOCK2 in WJ-MSCs did not affect cell stemness. Stemness was evaluated using flow cytometry, by identifying the expression of MSC-positive markers (CD44, CD73, CD90, CD105, and CD166). Expression of MSC-negative markers was confirmed using the hematopoietic markers (CD11b, CD14, CD19, CD34, CD45, and HLA-DR). Supplementary Figure 2: C2C12 cells were incubated in serum-free medium for 24 h to cause apoptosis. (a) The C2C12 cells were cultured with or without FBS for 24 h. (b) The apoptosis markers were upregulated in C2C12 cultured without FBS. Normalized protein levels indicate the value converted based on the FBS (-) group. (c) Representative fluorescence images showed apoptosis induction due to serum deficiency in C2C12 (green, apoptotic cells; blue, viable cells). The chart quantified the apoptotic rate of each group. Data are shown as means ± SEM. Two-tailed Student's t-test. ∗∗∗p < 0.001 and ∗∗p < 0.01. Supplementary Figure 3: residual cell number and antifibrotic effect according to the differences in AURKA mRNA expression of WJ-MSCs in the skeletal muscle of mdx mice. (a) The number of residual WJ-MSCs in mouse leg muscle (gastrocnemius muscle and thigh muscle) was identified using human Alu using real-time PCR. (b) Protein expression levels of fibronectin were confirmed using western blot. Normalized protein level indicates the value converted based on the mdx control group. One-way ANOVA with Tukey's multiple comparison test. (c) Fibrotic area was confirmed based on collagen deposition through Sirius Red staining of the gastrocnemius muscles of mdx mice. Data are shown as means ± SEM. One-way ANOVA with Duncan's multiple range test (a) and Tukey's multiple comparisons test (b, c). ∗∗∗p < 0.001, ∗∗p < 0.01, and ∗p < 0.05. [file 4711499.f1.zip › Supplementary figures.docx]

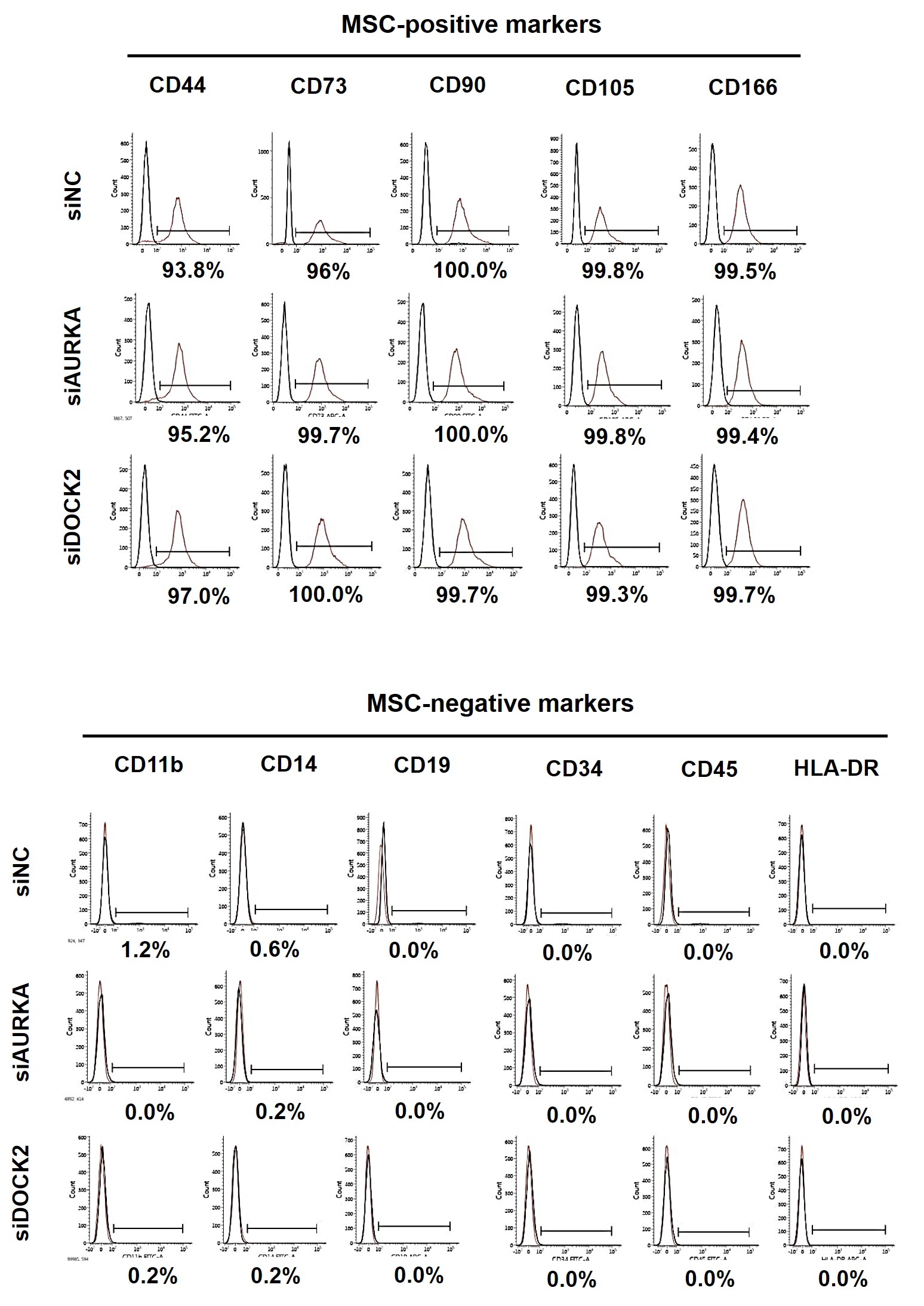


Supplementary Figure 1. siRNA transfection of AURKA and DOCK2 in WJ-MSCs did not affect cell stemness. Stemness was evaluated using flow cytometry, by identifying the expression of MSC-positive markers (CD44, CD73, CD90, CD105, and CD166). Expression of MSC-negative markers was confirmed using the hematopoietic markers (CD11b, CD14, CD19, CD34, CD45, and HLA-DR).


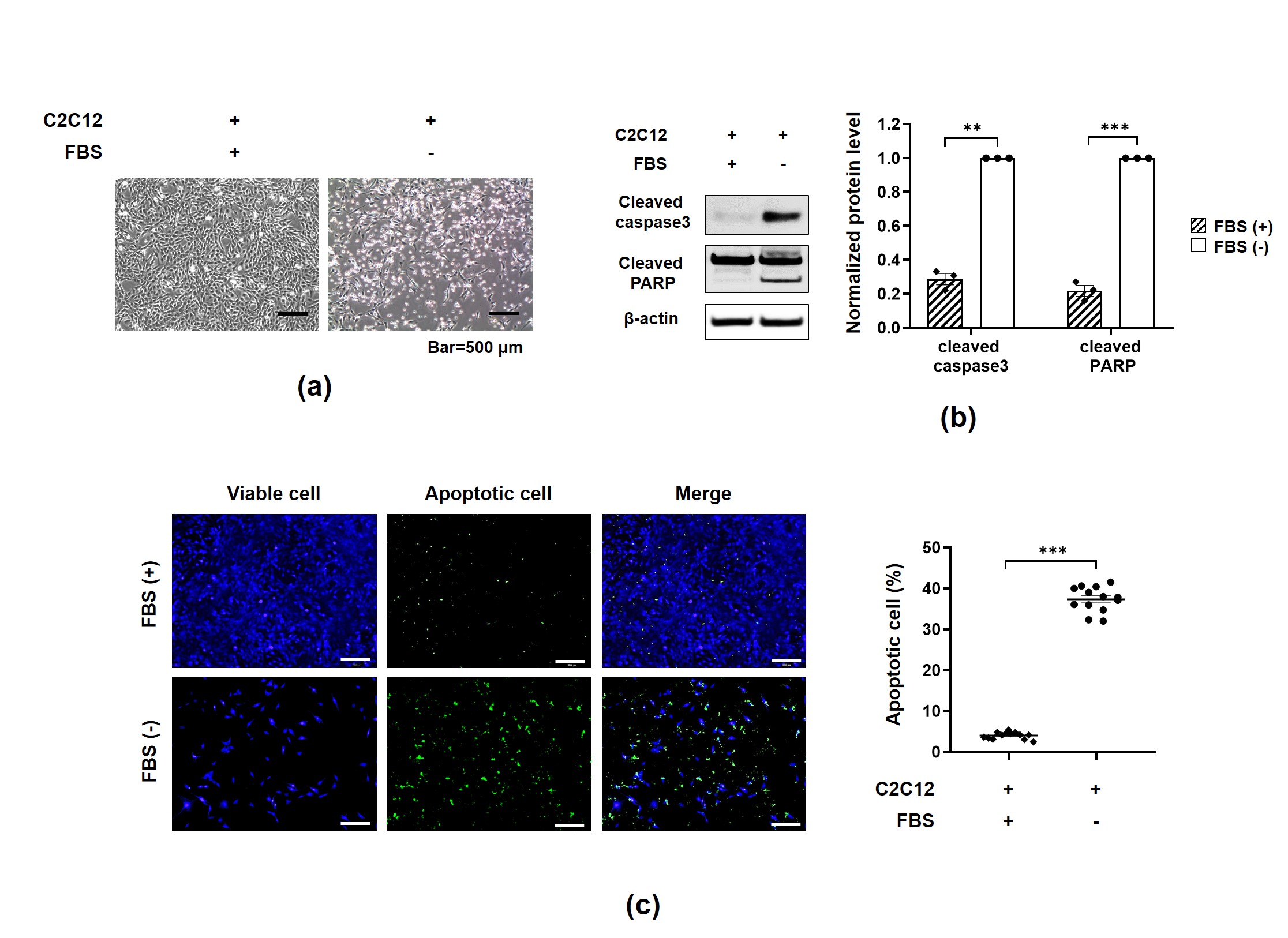


Supplementary Figure 2. C2C12 cells were incubated in serum-free medium for 24 h to cause apoptosis. (a) The C2C12 cells were cultured with or without FBS for 24 h. (b) The apoptosis markers were upregulated in C2C12 cultured without FBS. Normalized protein levels indicate the value converted based on the FBS (-) group. (c) Representative fluorescence images showed apoptosis induction due to serum deficiency in C2C12 (green, apoptotic cells; blue, viable cells). The chart quantified the apoptotic rate of each group. Data are shown as means ± SEM. Two-tailed Student’s t- test. ***p < 0.001 and **p < 0.01.


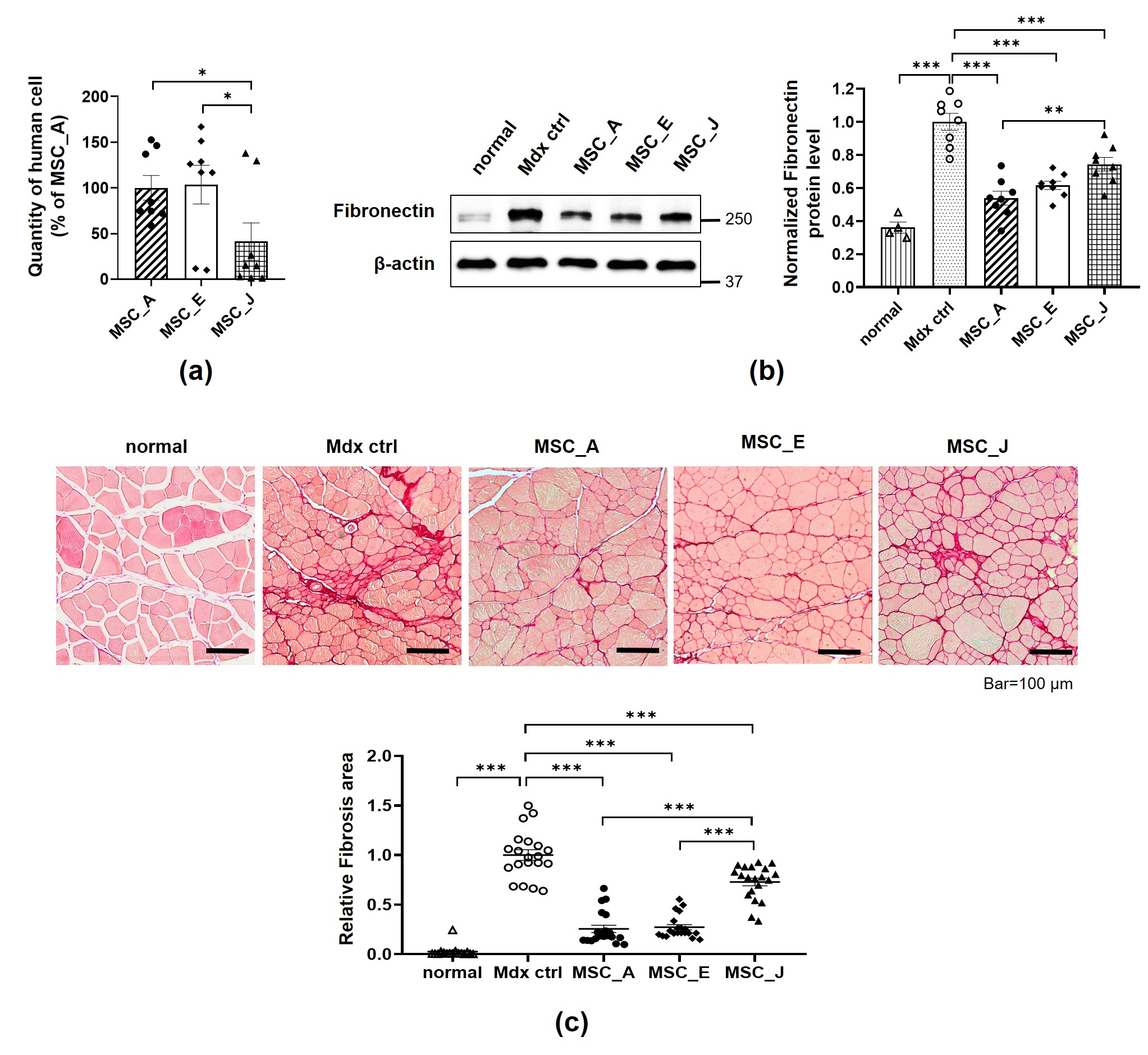


Supplementary Figure 3. Residual cell number and anti-fibrotic effect according to the differences in AURKA mRNA expression of WJ-MSCs in the skeletal muscle of mdx mice. (a) The number of residual WJ-MSCs in mouse leg muscle (gastrocnemius muscle and thigh muscle) was identified using human Alu using real-time PCR. (b) Protein expression levels of fibronectin were confirmed using western blot. Normalized protein level indicates the value converted based on the mdx control group. One-way ANOVA with Tukey’s multiple comparisons test. (c) Fibrotic area was confirmed based on collagen deposition through Sirius Red staining of the gastrocnemius muscles of mdx mice. Data are shown as means ± SEM. One-way ANOVA with Duncan’s multiple range test (a) and Tukey’s multiple comparisons test (b, c). ***p < 0.001, **p < 0.01, and *p < 0.05.
